# Assessing Sex Differences in the Risk of Cardiovascular Disease and Mortality per Increment in Systolic Blood Pressure: A Systematic Review and Meta-Analysis of Follow-Up Studies in the United States

**DOI:** 10.1371/journal.pone.0170218

**Published:** 2017-01-25

**Authors:** Yu-Chung Wei, Nysia I. George, Ching-Wei Chang, Karen A. Hicks

**Affiliations:** 1 Division of Bioinformatics and Biostatistics, National Center for Toxicological Research, United States Food and Drug Administration, Jefferson, Arkansas, United States of America; 2 Department of Statistics, Feng Chia University, Taichung, Taiwan; 3 Genentech, Inc., South San Francisco, California, United States of America; 4 Office of Drug Evaluation I, Division of Cardiovascular and Renal Products, Center for Drug Evaluation and Research, United States Food and Drug Administration, Silver Spring, Maryland, United States of America; Hunter College, UNITED STATES

## Abstract

In the United States (US), cardiovascular (CV) disease accounts for nearly 20% of national health care expenses. Since costs are expected to increase with the aging population, informative research is necessary to address the growing burden of CV disease and sex-related differences in diagnosis, treatment, and outcomes. Hypertension is a major risk factor for CV disease and mortality. To evaluate whether there are sex-related differences in the effect of systolic blood pressure (SBP) on the risk of CV disease and mortality, we performed a systematic review and meta-analysis. We conducted a comprehensive search using PubMed and Google Scholar to identify US-based studies published prior to 31 December, 2015. We identified eight publications for CV disease risk, which provided 9 female and 8 male effect size (ES) observations. We also identified twelve publications for CV mortality, which provided 10 female and 18 male ES estimates. Our meta-analysis estimated that the pooled ES for increased risk of CV disease per 10 mmHg increment in SBP was 25% for women (95% Confidence Interval (CI): 1.18, 1.32) and 15% for men (95% CI: 1.11, 1.19). The pooled increase in CV mortality per 10 mm Hg SBP increment was similar for both women and men (Women: 1.16; 95% CI: 1.10, 1.23; Men: 1.17; 95% CI: 1.12, 1.22). After adjusting for age and baseline SBP, the results demonstrated that the risk of CV disease per 10 mm Hg SBP increment for women was 1.1-fold higher than men (*P*<0.01; 95% CI: 1.04, 1.17). Heterogeneity was moderate but significant. There was no significant sex difference in CV mortality.

## Introduction

Cardiovascular (CV) disease is a worldwide health problem. However, compared to other high-income countries, the United States (US) spends significantly more on healthcare yet ranks lower in many outcome measures of health, including average life expectancy [1]. In the US, CV disease is the leading cause of death [2], and treatment of CV disease accounts for approximately 1/6^th^ of the healthcare budget [3]. Approximately one in every five CV deaths is attributed to hypertension [4, 5]. Projections show that by 2030, the prevalence of CV disease and hypertension in the US will be 43.9% and 41.4%, respectively [4]. Compared to other populations, the US population has unique healthcare challenges given the epidemic of overweight and obesity, which predisposes adults and children to hypertension and other cardiovascular risk factors [6]. In addition, data from self-report surveys indicated that the age-adjusted prevalence of heart disease and stroke for both men and women was highest in the US in comparison to 12 European countries [7]. Thus, understanding the relationship between hypertension and the risk of CV disease and mortality in the US population is critical in directing efforts to improve public health.

Numerous US population-based studies have demonstrated an association between hypertension and the risk of CV disease and mortality, but there is no consensus on whether there are sex-related differences in these risks. Studies have either failed to investigate sex differences, even if investigators acknowledged that these differences existed, or evaluated sex-specific differences but reported inconsistent findings [8–14]. For example, previous studies suggested that women had a higher risk for CV disease than men, although there were no significant sex-related differences in blood pressure (BP) [10]. In Vasan et *al*., the incidence of CV disease in subjects with prehypertension was higher in men than in women for all age groups; however, women had higher hazard ratios than men when comparing the risk of CV disease between prehypertensives and normotensives [11]. Generally, analyses based on observational studies have indicated that CV mortality rates are higher in men [12, 13], but in Seeman et *al*., the odds ratio for mortality due to coronary heart disease (the largest contributor to CV mortality) differed by sex when particular stages of hypertension were compared to the baseline normotensive group [14]. Whether there are sex-related differences in myocardial infarction mortality rates based on blood pressure is also unclear [15–19].

Overall, hypertension affects more men than women. Through 65 years of age, the percentage of men with hypertension is similar to or higher than the percentage of age-matched women with hypertension [20]. After age 65, more women than men have hypertension [21]. Although clinical studies have shown that, on average, BP is higher in men than in women, women have worse outcomes after a CV event [22, 23]. Given the inconsistent findings to date with respect to sex-related BP differences and CV outcomes, we conducted a meta-regression analysis to assess whether there was a significant sex difference in the risk of CV disease (and mortality) per 10 mm Hg increment in systolic blood pressure (SBP). Our analysis focused on SBP since it is a strong predictor for CV disease [24, 25].

## Materials and Methods

### Search strategy and study selection

We performed a systematic review in accordance with the *Meta-Analysis of Observational Studies in Epidemiology* [26] and the Preferred Reporting Items for Systematic Reviews and Meta-Analyses (PRISMA) statement checklist guidelines [27] (S1 Table). We considered publications identified through PubMed and Google Scholar that were published before 31 December, 2015 and excluded publications that were not written in English. The list of search terms included cardiovascular disease, heart disease, blood pressure, systolic blood pressure, mortality, death, risk, sex factors, risk factors, men, and female. Additional search terms were necessary for Google Scholar to narrow down the numbers of results. The additional terms included baseline, follow up, relative risk, odds ratio, hazard ratio, standard deviation, SD, confidence interval, and CI. The syntax for both database searches is provided in S2 Table.

We used the following criteria to identify publications for extensive review: 1) specific outcomes were measured in the US population; 2) mixed race studies were permitted, but cross-country (e.g., US and Europe) studies were not allowed; 3) specific outcomes and descriptive statistics were reported separately for men and women; 4) baseline mean or median SBP was reported for studies that estimated the risk of CV disease per a specified mm Hg increase in SBP or range of BP values was reported for studies reporting the risk of CV disease for the comparison of different BP categories to a reference group; 5) the endpoint event was CV disease (e.g., coronary heart disease, ischemic heart disease, or myocardial infarction); 6) an effect size (ES) such as relative risk (RR), odds ratio (OR), or hazard ratio (HR) and the corresponding standard error (SE) and/or confidence interval (CI) was reported for risk of CV disease or mortality per a given mm Hg increase in SBP. Publications that included ESs between categorized blood pressure groups were also permitted. Two independent reviewers searched for and subsequently assessed each full-text publication. General quality assessment of each study followed the quality ratings guidelines outlined by the Newcastle-Ottawa Quality Assessment Scale for cohort studies [28].

### Data extraction and synthesis

The majority of ES estimates gathered from each study were adjusted HR estimates; however, a few ESs were adjusted RR and OR estimates (S3 and S4 Tables). RR is a type of HR with no time unit and can approximate the OR in studies with low event rates [29]. Thus, all three estimates (HR, RR, and OR) were treated as equivalent indices of risk in this study [30, 31]. Study characteristics, such as mean SBP at baseline (SBP_B_), sample size, and sex, were recorded for each publication. All studies reported clinic BP measurements. It should be noted that measurements of BP in the Physician’s Heart Study and the Women’s Health Study were self-reported by physicians and registered nurses, respectively [32]. The authors cite findings by Klag et *al*. [33] and Ascherio et *al*. [34] to highlight high correlation between self-reported and measured blood pressure in physicians and health professionals, respectively. With the exception of one cohort, all studies indicated that BP was measured while the subject was seated; blood pressure in the Chicago Heart Association Detection Project in Industry was measured in the supine position. Where possible, data for other CV risk factors were also collected, including age, follow-up years, race, body mass index, smoking status, cholesterol level, and history of diabetes.

Publications selected for this study reported their findings using two different analytical methods. Some studies reported an ES for CV disease risk per a given unit increase in SBP. Other studies reported an ES between categorized groups; each study used a normotensive group as the reference. For the former group of studies, we adjusted all ESs to reflect a 10 mm Hg increment in SBP and used a log-linear transformation [9] to recalculate ESs and their corresponding standard errors and CIs. For the latter group of studies, we computed the unit of SBP increment as the difference between baseline SBP values for the case and normotensive group. The ES of each categorized group was recalculated for 10 mm Hg increments using the same transformation method mentioned above.

Some accommodations were necessary to standardize the effect size and corresponding baseline information across studies. First, some studies reported findings for a category, which was described by an interval of values. In these cases, it was necessary to summarize the category using a single numeric value. The midpoint of the interval was computed when the boundary of the interval was provided. However, for half-bounded intervals with no lower bound, the width of the adjacent category was used to compute the midpoint of the half-bounded interval. For example, if a study reported an odds ratio for two SBP groups: 140–159 mm Hg and <140 mm Hg, then the baseline SBP was set to 150 and 130 for the respective groups [14]. Second, if baseline information was reported for several subgroups but only one ES was reported for the entire study, then a weighted mean was used to summarize baseline information. Lastly, in the event that multiple publications referred to the same study, we selected the publication with the largest sample size. Excluded publications due to overlapping studies are listed in S5 Table.

### Statistical analysis

We used a random-effects meta-analysis model to evaluate sex differences in the risk of CV disease and mortality. Each study was weighted by the inverse variance method. Multiple regression moderator analysis was utilized to test whether potential moderator variables significantly affected the risk of CV disease (and mortality) per 10 mm Hg SBP increment (evaluated at α = 0.05). We considered the full model, which included all main effects of the considered moderator variables, and the resulting subset models. Analyses for the full and the optimal subset model (model with the lowest Bayesian Information Criterion (BIC)) are reported [35].

The level of heterogeneity was measured by Cochran’s Q test [36] and I^2^, where values of 25%, 50%, and 75% corresponded to low, moderate, and high evidence of heterogeneity [37]. We assessed publication bias through visual inspection of the funnel plot. We also performed Begg and Mazumdar’s rank correlation test (Begg’s test) [38] and Egger’s regression test (Egger’s test) [39] to quantify bias demonstrated in the funnel plot. The influence of a specific ES observation on the overall results was investigated with leave-one-out sensitivity analysis. All analyses were performed using the *metafor* package in R project (http://r-project.org).

## Results

**Fig 1:**
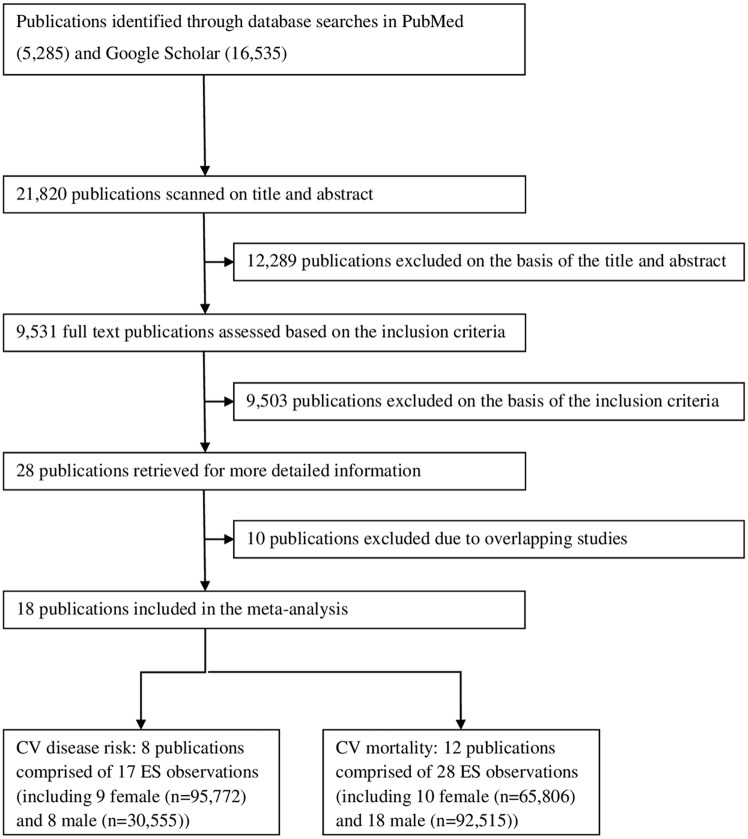
Flow chart of publication selection process.

Eighteen publications met the selection and inclusion criteria. Two of the 18 publications discussed the risk of both CV disease and CV mortality. The resulting data for the risk of CV disease included 9 female and 8 male ES observations (totaling 95,772 female and 30,555 male subjects, respectively) [11, 14, 32, 40–44]. Likewise, the CV mortality data consisted of 10 female and 18 male ES estimates (totaling 65,806 female and 92,515 male subjects, respectively) [13, 14, 43, 45–53]. A flowchart of the data search and selection process is provided in Fig 1. A summary of the studies for the risk of CV disease is presented in Table 1 and S3 Table; the studies for CV mortality are presented in Table 2 and S4 Table.

**Table 1:** Characteristics of CV disease risk studies.

	Study^a^	ES ID^b^	Sample Size	Reported ES [95% CI]	Unit (mm Hg)	SBP_B_ (mm Hg)	Age (year)	Follow-up (year)
***Female***	ARIC[40]	R-F-ARIC	7301	1.38 [1.26, 1.51]	20.00	120.00	57.00	11.00
	CHS[41]	R-F-CHS	2979	1.13 [1.04, 1.23]	10.00	136.82	72.42	4.80
	EPESE[14]	R-F-EPESE	436	1.42 [0.64, 3.17]	20.00	150.00	74.90	6.00
	FHS[11]	R-F-FHS-1	1126	1.50 [0.90, 2.50]	14.00	122.00	51.00	10.00
		R-F-FHS-2	891	2.50 [1.60, 4.10]	24.00	132.00	55.00	10.00
	FOS[42]	R-F-FOS	1312	1.01 [1.00, 1.02]	1.00	123.30	54.20	14.00
	WHI[43]	R-F-WHI-1	23596	1.66 [1.44, 1.92]	21.00	130.00	62.60	7.70
		R-F-WHI-2	21187	2.89 [2.52, 3.32]	32.00	141.00	64.50	7.70
	WHS[32]	R-F-WHS	36944	1.30 [1.22, 1.38]	10.00	124.00	53.80	6.00
***Male***	ARIC[40]	R-M-ARIC	5533	1.26 [1.15, 1.39]	20.00	122.00	57.00	11.00
	CHS[41]	R-M-CHS	1967	1.11 [1.04, 1.18]	10.00	135.98	73.02	4.80
	EPESE[14]	R-M-EPESE	284	1.29 [0.57, 2.91]	20.00	150.00	73.90	6.00
	FHS[11]	R-M-FHS-1	1059	1.30 [1.00, 1.90]	11.00	122.00	49.00	10.00
		R-M-FHS-2	903	1.60 [1.10, 2.20]	20.00	131.00	51.00	10.00
	FOS[42]	R-M-FOS	1047	1.01 [1.00, 1.02]	1.00	128.30	54.50	14.00
	PHS[32]	R-M-PHS	17862	1.20 [1.16, 1.24]	10.00	126.10	53.20	13.00
	Other[44]	R-M-OT	1900	1.42 [1.05, 1.94]	17.50	150.00	52.00	3.80

CI: confidence interval; CV: cardiovascular; ES: effect size; ID: identification; SBP_B_: mean systolic blood pressure at baseline

^a^Abbreviation of study names: ARIC: Atherosclerosis Risk In Communities Study; CHS: Cardiovascular Health Study; EPESE: Epidemiologic Studies of the Elderly; FHS: Framingham Heart Study; FOS: Framingham Offspring Study; PHS: Physician’s Health Study; WHI: Women’s Health Initiative; WHS: Women’s Health Study.

^b^Each ES ID is denoted by a string consisting of an R for risk, “-sex abbreviation (F for female; M for male)” and “-study abbreviation”. If there were more than two ES estimates from the same study, a numbered list was added to the ES ID.

**Table 2:** Characteristics of CV mortality studies.

	Study^a^	ES ID^b^	Sample Size	Reported ES [95% CI]	Unit (mm Hg)	SBP_B_ (mm Hg)	Age (year)	Follow-up (year)
***Female***	CHA[45]	M-F-CHA-1	6909	1.32 [1.22, 1.43]	19.50	135.00	49.30	25.00
		M-F-CHA-2	1013	1.24 [1.10, 1.40]	21.30	146.60	63.10	25.00
	*CHA[46]	M-F-CHA-3	7302	1.10 [0.91, 1.34]	15.00	123.24	26.76	31.00
	CHHS[13]	M-F-CHHS-1	741	1.66 [1.31, 2.10]	28.56	139.20	50.10	30.00
		M-F-CHHS-2	454	1.30 [1.02, 1.65]	28.56	161.70	50.40	30.00
	EPESE[14]	M-F-EPESE	491	1.07 [0.55, 2.09]	20.00	150.00	74.90	6.00
	HERS[47]	M-F-HERS	2762	1.15 [1.02, 1.30]	19.00	135.00	66.00	4.10
	RBS[48]	M-F-RBS	1351	1.23 [1.10, 1.38]	10.00	134.99	61.33	14.40
	WHI[43]	M-F-WHI-1	23596	1.58 [1.12, 2.21]	21.00	130.00	62.60	7.70
		M-F-WHI-2	21187	3.02 [2.18, 4.18]	32.00	141.00	64.50	7.70
***Male***	CHA[45]	M-M-CHA-1	10874	1.26 [1.12, 1.41]	15.20	134.40	29.70	25.00
		M-M-CHA-2	8307	1.26 [1.19, 1.33]	19.20	140.30	48.50	25.00
		M-M-CHA-3	1257	1.22 [1.11, 1.35]	21.50	150.00	63.00	25.00
	CHHS[13]	M-M-CHHS-1	653	1.28 [1.02, 1.60]	28.56	140.90	49.90	30.00
		M-M-CHHS-2	333	1.71 [1.27, 2.29]	28.56	152.70	49.90	30.00
	ECHS[49]	M-M-ECHS	322	1.02 [1.00, 1.03]	1.00	159.20	50.80	20.00
	EPESE[14]	M-M-EPESE	346	1.08 [0.54, 2.16]	20.00	150.00	73.90	6.00
	HAHS[50]	M-M-HAHS-1	9577	1.17 [1.03, 1.32]	14.80	123.40	18.40	56.30
		M-M-HAHS-2	2176	1.33 [1.14, 1.56]	27.90	136.50	18.50	56.30
		M-M-HAHS-3	380	1.63 [1.26, 2.12]	39.30	147.90	18.40	56.30
	PGC[51]	M-M-PGC	873	1.03 [1.01, 1.05]	1.00	131.70	51.10	19.00
	PHS[52]	M-M-PHS-1	23878	1.52 [1.08, 2.15]	10.00	122.10	44.40	5.70
		M-M-PHS-2	16797	1.56 [1.28, 1.89]	10.00	124.90	54.70	5.70
		M-M-PHS-3	9076	1.23 [1.05, 1.43]	10.00	128.60	64.30	5.70
		M-M-PHS-4	3412	1.14 [0.99, 1.31]	10.00	133.30	74.30	5.70
	RBS[48]	M-M-RBS	1100	1.09 [1.00, 1.18]	10.00	138.75	62.73	14.40
	WCG[53]	M-M-WCG-1	2249	1.34 [1.13, 1.60]	15.12	130.54	45.00	22.00
		M-M-WCG-2	905	1.58 [1.37, 1.82]	15.12	135.18	55.00	22.00

CI: confidence interval; CV: cardiovascular; ES: effect size; ID: identification; SBP_B_: mean systolic blood pressure at baseline

^a^Abbreviation of study names: CHA: Chicago Heart Association Detection Project in Industry; CHHS: Charleston Heart Study; ECHS: Evans County Heart Study; EPESE: Epidemiologic Studies of the Elderly; HAHS: Harvard Alumni Health Study; HERS: Heart and Estrogen/Progestin Replacement Study; PGC: Peoples Gas Company Study; PHS: Physician’s Health Study; RBS: Rancho Bernardo Study; WCG: Western collaborative group study; WHI: Women’s Health Initiative.

^b^Each ES ID is denoted by a string consisting of an M for mortality, “-sex abbreviation (F for female; M for male)” and “-study abbreviation”. If there were more than two ES estimates from the same study, a numbered list was added to the ES ID.

*Study CHA[46] differs from CHA[45] in that it assesses a different age population.

### Cardiovascular risk

**Fig 2:**
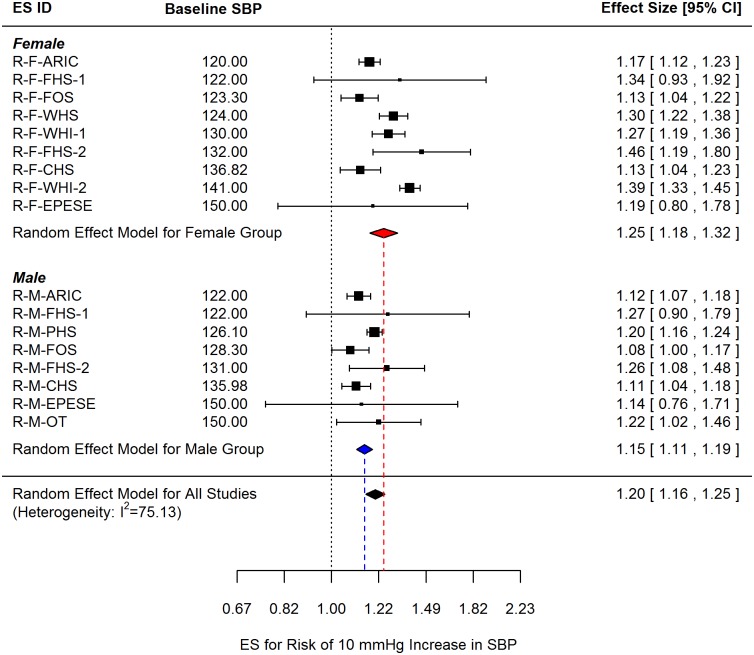
Sex-specific and overall effect sizes (ES) for CV disease risk per 10 mm Hg increment in SBP. ES observations are ordered by baseline SBP values. The corresponding ES IDs are listed in Table 1.

The pooled ES for CV disease risk per 10 mmHg increment in SBP was 1.20 (95% CI: 1.16, 1.25) (Fig 2), which indicates that 10 mmHg increments in SBP are significantly associated with an increased risk of CV disease for the US population. Independent analysis for both sex groups revealed that the pooled ES for women was 1.25 (95% CI: 1.18, 1.32) and the pooled ES for men was 1.15 (95% CI: 1.11, 1.19). However, since the 95% CIs for each subgroup overlapped, the pooled ES estimates were not significantly different in the absence of moderator variables. Heterogeneity in published estimates of the risk of CV disease associated with a 10 mm Hg increase in SBP was high (I^2^ = 75.13%). Based on the aforementioned findings, available explanatory variables with no missing data (i.e. sex, age, number of follow-up years, and baseline SBP) were evaluated as possible moderator variables.

Multiple meta-regression models were used to assess the significance of potential moderator variables. In the full model, the variable for follow-up years was the only moderator that did not significantly account for the variance in the risk of CV disease per 10 mm Hg increment in SBP. The optimal model with sex (*P*<0.01), age (*P* = 0.02), and baseline SBP (*P*<0.01) accounted for 59% of the variation in the risk of CV disease (Table 3). Each 10 mm Hg increase in SBP increased the risk of CV disease in women by approximately 1.1 times that for men by age and baseline SBP. Thus, women were affected more adversely per each 10 mm Hg increase in SBP. The observed increase in the risk of CV disease per SBP increment was estimated to diminish slightly with increasing age and is consistent with previous research [54]. After adjusting for the main effects of sex, age, and baseline SBP, heterogeneity was reduced to I^2^ = 48.81% (*P* = 0.01). Additionally, publication bias is not suspected as tests for funnel plot asymmetry were not significant (Begg’s test: *P* = 0.78; Egger’s test: *P* = 0.64). Although one ES observation fell on the 95% confidence band (S1 Fig), a sensitivity analysis showed that the meta-regression results were not significantly different after removing the potential outlier observation.

**Table 3:** Moderator estimators for CV disease risk and CV mortality.

	Model	Moderator	*e*^β^	[95% CI]	*P*
***CV Disease Risk***	Optimal	**Sex** (1:femele, 0:male)	1.10	[1.04, 1.17]	<0.01*
		**Age** (year)	0.99	[0.99, 1.00]	0.02*
		**SBP**_**B**_ (mmHg)	1.01	[1.00, 1.01]	<0.01*
	Full	**Sex** (1:femele, 0:male)	1.09	[1.02, 1.16]	<0.01*
		**Age** (year)	0.99	[0.99, 1.00]	<0.01*
		**SBP**_**B**_ (mmHg)	1.01	[1.00, 1.01]	0.03*
		**Follow-up** (year)	0.99	[0.98, 1.01]	0.31
***CV Mortality***	Optimal	**Follow-up** (year)	1.00	[1.00, 1.00]	0.06
	Full	**Sex** (1:femele, 0:male)	0.98	[0.92, 1.05]	0.58
		**Age** (year)	1.00	[0.99, 1.00]	0.36
		**SBP**_**B**_ (mmHg)	1.00	[1.00, 1.00]	0.68
		**Follow-up** (year)	1.00	[0.99, 1.00]	0.10

CI: confidence interval; CV: cardiovascular; SBP_B_: mean systolic blood pressure at baseline

*Significant (*P*<0.05)

### Cardiovascular mortality

**Fig 3:**
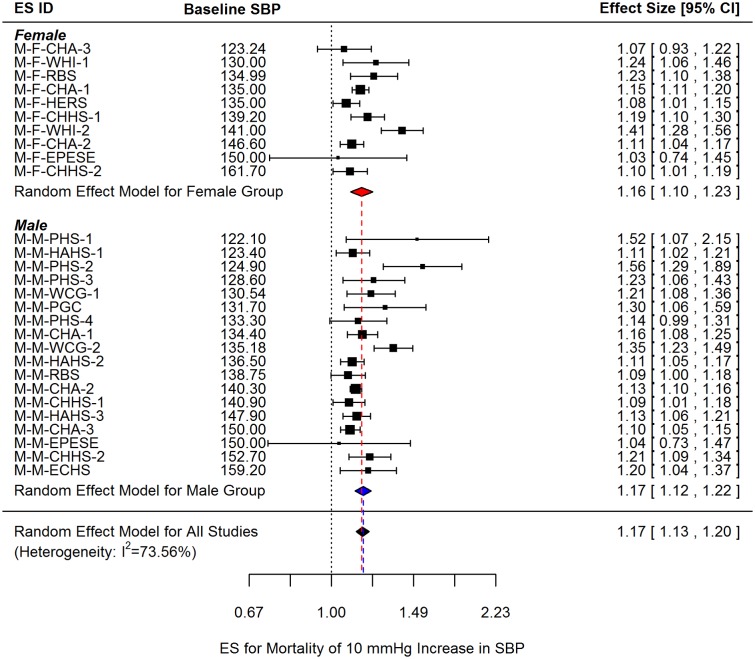
Sex-specific and overall effect sizes (ES) for CV mortality per 10 mm Hg increment in SBP. ES observations are ordered by baseline SBP values. The corresponding ES IDs are listed in Table 2.

The pooled ES estimate for CV mortality per 10 mm Hg SBP increment was 1.17 (95% CI: 1.13, 1.20) (Fig 3). Independent results for sex-specific analysis revealed that the pooled ES for women (1.16; 95% CI: 1.10, 1.23) and men (1.17; 95% CI: 1.12, 1.22) were not significantly different. High between-study heterogeneity was observed by I^2^ (73.56%). Similar to the risk of CV disease, sex, age, follow-up years, and baseline SBP were examined as possible moderator variables.

Multiple meta-regression analysis of the full model showed that sex (*P* = 0.58), age (*P* = 0.36), baseline SBP (*P* = 0.68), and follow-up years (*P* = 0.10) were not significant moderators for mortality, as noted in previous work [13, 48, 55]. Each 10 mm Hg in SBP increased CV mortality by approximately 16–17% for both women and men (Table 3). The optimal model for CV mortality included the main effect of only follow-up years; however, follow-up years was still not a significant main effect (*P* = 0.06). Thus, consideration of a meta-regression model with moderator variables did not effectively reduce heterogeneity (I^2^ = 69.83%). Unfortunately, results of the funnel plot asymmetry testing were inconsistent: Begg’s test was significant (*P* = 0.01) but Egger’s test was not (*P* = 0.10). Two ES observations fell outside of the 95% confidence bands (S2 Fig). However, a sensitivity analysis showed that the ES estimates were not influential observations.

## Discussion

The association between BP and the risk of CV disease and CV mortality has been demonstrated previously, but the influence of moderators is still unclear. In particular, research regarding the significance of sex in evaluating the risk of CV disease and CV mortality has been inconclusive. Some studies indicated that the risk of CV disease was higher in women than men for the same unit change in SBP or within the same BP category [10, 11] while other studies reported no significant findings [32, 56]. Inconsistent results were also observed in the analysis of CV mortality [13, 48, 55]. Furthermore, a meta-analysis of individual data from 9,357 European, Asian, and South American subjects from 11 populations demonstrated that while the hazard ratios associated with nighttime SBP were significantly higher in women than in men, the hazard ratios associated with conventional BP measurements showed no sex differences [57]. Similar conclusions were reached in a separate meta-analysis. Roush et *al*. [58] conducted a meta-analysis of 10 cohorts consisting of patients from Europe, Brazil, and Japan to assess the interaction between sex and SBP. They concluded that a 1 SD (and generally, a 10 mm Hg) increase in ambulatory SBP (but not clinic SBP) demonstrated significantly higher risks for cardiovascular events in women than in men.

In contrast, findings from our meta-regression analysis of US population-based studies indicate that women experienced a 10% greater risk in CV disease per 10 mm Hg increment in SBP than men, after adjusting for age and baseline SBP. It is important to note that meta-regression analyses may suffer from certain biases due to the use of study-level covariates to characterize moderator variables. Although moderator variables could be attenuated by measurement error, this was a minor issue in our work since the sample size of each study was sufficient. The relationship between risk and proportion or mean estimates across studies may also not mirror that of individual data within a study; this is commonly referred to as the ecological fallacy [59]. Finally, the effect of moderators may be better interpreted when the characteristic being studied as high variance across studies. Even if a covariate has high variability within a study and is significant, if mean estimates across studies are similar, then the covariate will be of limited use in meta-regression. Furthermore, unlike our meta-regression analysis, the Boggia et *al*. [57] and Roush et *al*. [58] meta-analyses were comprised of studies that provided both a female and male risk estimate. Thus, we carried out an ancillary meta-analysis using only the six studies that provided CV disease risk estimates for both sexes (i.e. studies with an ES ID ending in ARIC, CHS, EPESE, FHS-1, FHS-2, and FOS as reported in Table 1). Based on this analysis, the increase in the risk of CV disease per 10 mm Hg SBP increment in women relative to men was 1.06 (*P* = 0.002; 95% CI: 1.02, 1.09). Heterogeneity was moderate and not significant (I^2^ = 40.61%; *P* = 0.17). Overall, our findings from both types of analysis are consistent with previous studies which demonstrated that women have worse outcomes after a cardiovascular event.

Although other meta-analyses have been conducted in this field [56], the present work is important for a number of reasons. First, we focused entirely on data pertaining to the US population, given the epidemic of overweight and obesity which impacts the development and treatment of hypertension and other cardiovascular risk factors. Currently, research efforts are being directed towards reducing myocardial infarctions (MI), heart failure (HF), and stroke rates in the United States and globally. For example, the Systolic Blood Pressure Intervention Trial (SPRINT) [60], a National Institutes of Health study scheduled to conclude in 2017, released preliminary findings suggesting that systolic blood pressure targets for cardiovascular risk should be lowered to achieve better health outcomes. Although our work does not address SBP goals, it does offer some insight on sex-based discrepancies that may influence the rates of CV disease. Second, since the meta-analysis model was constructed to assess CV events for continuous measures of SBP, the subsequent inference may be more precise. In order to preserve as much information as possible, we used a simple statistical method to transform the categorized responses to continuous measurements. Some meta-analysis studies derived continuous measures from categorized data via dose-response methods [61], which focus on trend estimation rather than response transformation. Since this particular method cannot aggregate categorized and continuous data structures, it was not suitable for this work. If the number of studies for each data type was large enough, separate analyses could have been performed for both data types. Third, prior investigations examined female-male data from the same study to assess sex-specific changes in the risk of CV disease per unit increase in SBP [56]. Peters et *al*.[56] used the relative risk ratio of women to men as the response variable, but the estimated response did not consider differences between the sexes such as sample size, age, and race. Thus, the estimated relative risk ratio may be misleading due to noise. Moreover, based on their analysis, it is difficult to determine whether the variation in relative risk ratio was due to sex, SBP, or risk of CV disease.

Providing a rationale for the lack of a sex-based difference in the association between SBP and CV mortality is complex. Cardiovascular mortality is not only influenced by demographic factors such as sex, age, CV risk factors, and comorbid conditions but also by treatment factors (e.g., medications to treat hypertension, hyperlipidemia, diabetes, heart failure, coronary artery disease, stroke, and peripheral vascular disease). This may explain the substantial heterogeneity in CV mortality despite the fact that major risk factors, including sex, age, baseline SBP, and follow-up years, were considered as possible moderators in the meta-regression model. Although none of the moderator variables available for this study could account for the changes in CV mortality associated with a 10 mm Hg increment in SBP, these variables may still affect CV mortality. The lack of significance may be because ES estimates obtained from each publication had already been adjusted for these major risk factors.

Our meta-analysis provides the most thorough evaluation of sex differences in the risk of CV disease and mortality per 10 mm Hg SBP increment for the US population. However, our work is not without limitations. Integrating studies with different types of data and with ESs that were adjusted for different SBP increments is an inherent limitation. In addition, there is an imbalance in total sample size for female and male subjects across studies. The imbalance is more prominent for cardiovascular disease risk and primarily due to the inclusion of two large female studies—the Women’s Health Initiative and the Women’s Health Study. Given the influence of adequately powered studies on meta-analysis, we included estimates from these studies. Another limiting factor was that risk factors were not adjusted in a consistent way across studies due to varying study designs. Unfortunately, due to missing data, we were unable to evaluate the influence of additional moderator variables such as race, smoking, BMI, obesity, and, diabetes. There is no good way to handle missing data. It is not easy to impute values for missing data when there are a limited number of observations from each study. Deleting studies with missing or inconsistent information would have resulted in low power due to an insufficient sample size. Although race is a significant factor in the relationship between SBP and CV disease [13], there was a wide variability in how race was reported in publications (percentage of a single specific race, percentage of two specific races, or no account of race) [14, 40, 41, 43, 44, 46, 47]. Therefore, it was impossible to aggregate the information in a consistent format to explore the effect of race in a meaningful way. Fortunately, many researchers adjusted for the race effect in their original analyses. Lastly, due to sample size limitations of data pertaining to the US population, all endpoints for CV events (e.g., CV disease, coronary heart disease, stroke, and myocardial infarction) were combined to provide a general assessment of sex-specific effects.

Despite the aforementioned limitations, this study demonstrated that the risk of CV disease per 10 mm Hg in SBP was higher in women than in men, adjusting for age and baseline SBP. In future studies, it may be reasonable to assess whether blood pressure targets for clinic CV risk assessment (e.g. clinic or 24-hr SBP) should be sex-based and thereby improve CV outcomes.

## Supporting Information

S1 Fig Funnel plot of meta-analysis for CV disease risk. The effect size observation R-F-FOS lies on the 95% confidence band.
(PDF)

S2 Fig Funnel plot of meta-analysis for CV mortality. The effect size observations M-F-WHI-2 and M-M-PHS-2 fell outside the 95% confidence band and two observations (M-F-HERS and M-M-WCG-2) lie on the confidence band.
(PDF)

S1 Table PRISMA checklist. (PDF)

S2 Table Search strategies for literature review. (PDF)

S3 Table Summary of studies for CV disease risk. (PDF)

S4 Table Summary of studies for CV mortality. (PDF)

S5 Table Excluded publications due to overlapping studies. (PDF)
