# Supplementary figures and images for: Assessing Sex Differences in the Risk of Cardiovascular Disease and Mortality per Increment in Systolic Blood Pressure: A Systematic Review and Meta-Analysis of Follow-Up Studies in the United States
Source: PLoS One. 2017 Jan 25;12(1):e0170218. doi: 10.1371/journal.pone.0170218 (PMC5266379; doi:10.1371/journal.pone.0170218)

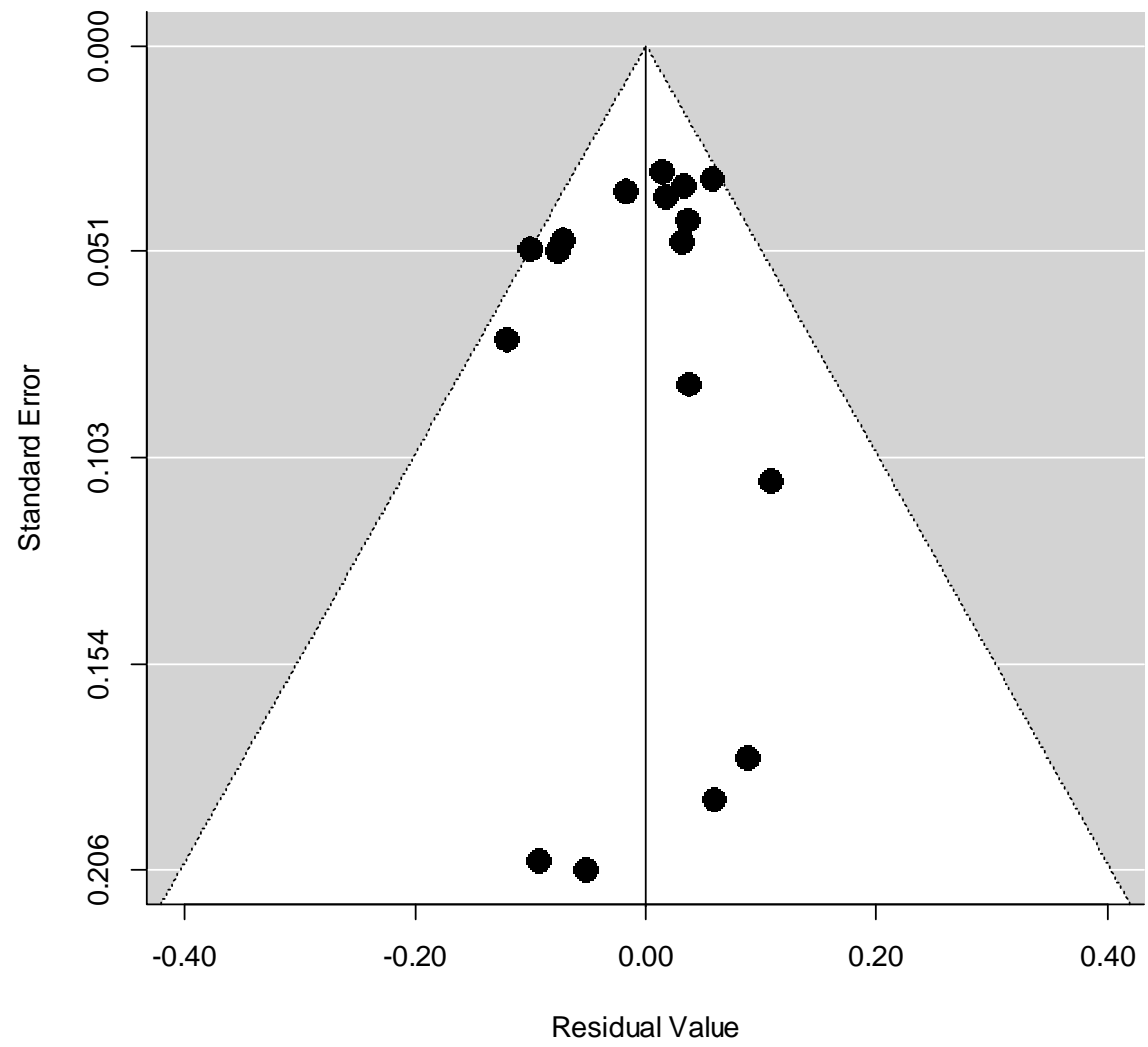

Supplement: S1 Fig — The effect size observation R-F-FOS lies on the 95% confidence band. (PDF) [file pone.0170218.s001.pdf]

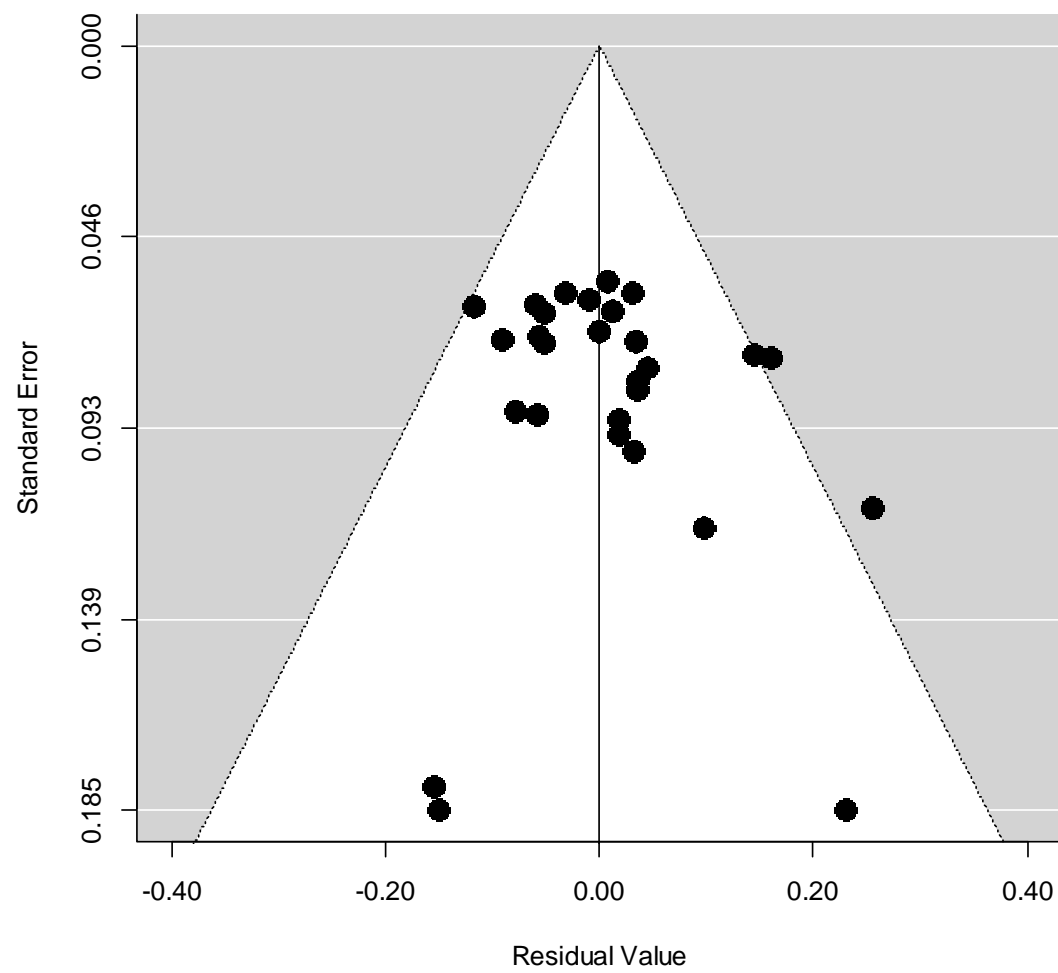

Supplement: S2 Fig — The effect size observations M-F-WHI-2 and M-M-PHS-2 fell outside the 95% confidence band and two observations (M-F-HERS and M-M-WCG-2) lie on the confidence band. (PDF) [file pone.0170218.s002.pdf]
