# Supplementary material for: Assessing Sex Differences in the Risk of Cardiovascular Disease and Mortality per Increment in Systolic Blood Pressure: A Systematic Review and Meta-Analysis of Follow-Up Studies in the United States
Source: PLoS One. 2017 Jan 25;12(1):e0170218. doi: 10.1371/journal.pone.0170218 (PMC5266379; doi:10.1371/journal.pone.0170218)
Supplement: S2 Table — (PDF) [file pone.0170218.s004.pdf]

S2 Table. Search strategies for literature review

Database	Date	Search	Records
PubMed	31 December, 2015	(("cardiovascular disease"[All Fields] OR "heart disease"[All Fields]) AND ("blood pressure"[All Fields] OR "blood pressure"[MeSH Terms]) AND("mortality"[All Fields] OR "mortality"[MeSH Terms] OR "death"[MeSH Terms]) AND ("0001/01/01"[PDAT] : "2016/06/30"[PDAT]) AND ("humans"[MeSH Terms] AND English[lang]) AND "loattrfull text"[sb]) OR (("cardiovascular disease"[All Fields] OR "heart disease"[All Fields]) AND ("blood pressure"[All Fields] OR "blood pressure"[MeSH Terms]) AND ("risk"[MeSH Terms]) AND ("sex factors"[MeSH Terms]) AND ("0001/01/01"[PDAT] : "2016/06/30"[PDAT]) AND ("humans"[MeSH Terms] AND English[lang]))	5,285
Google Scholar	31 December, 2015	"baseline" "risk factors" "heart disease" "follow up" "systolic blood pressure" ("men" OR "women") ("mortality" OR "risk") ("relative risk" OR "odds ratio" OR "hazard ratio") ("SD" OR "standard deviation" OR "CI" OR "confidence interval")	16,535
