# Supplementary material for: Assessing Sex Differences in the Risk of Cardiovascular Disease and Mortality per Increment in Systolic Blood Pressure: A Systematic Review and Meta-Analysis of Follow-Up Studies in the United States
Source: PLoS One. 2017 Jan 25;12(1):e0170218. doi: 10.1371/journal.pone.0170218 (PMC5266379; doi:10.1371/journal.pone.0170218)
Supplement: S3 Table — (PDF) [file pone.0170218.s005.pdf]

S3 Table. Summary of Studies for CV Disease Risk.

	Study[a]	Disease[b]	ES type[c]	Adjustment Variables
Female				
	ARIC[40]	CHD	HR	Age, African-American, Cholesterol medication, Current smoker, Diabetic, HDL cholesterol, Hypertension medication, Total cholesterol
	CHS[41]	MI	RR	Age, Clinical CVD, Glucose, Race
	EPESE[14]	MI	OR	Age, Antihypertensive medication, BMI, Chest pain on exertion, Diabetes, Prevalent CHD at baseline, Smoking,
	FHS[11]	CVD	HR	Age, BMI, Diabetes mellitus, Smoking status, Total cholesterol
	FOS[42]	CVD	HR	Age, BMI, Cholesterol, HDL Cholesterol, HbA1c, Smoking, Diabetes
	WHI[43]	CVD	HR	Age, BMI, Diabetes mellitus, Current smoking, High cholesterol
	WHS[32]	CHD	RR	Age, Alcohol intake, BMI, Current hypertension treatment, Diabetes, Exercise, Parental history if MI before 60y, Smoking status, Randomized aspirin treatment, Randomized beta-carotene treatment, Randomized vitamin E treatment
Male				
	ARIC[40]	CHD	HR	Age, African-American, Cholesterol medication, Current smoker, Diabetic, HDL cholesterol, Hypertension medication, Total cholesterol
	CHS[41]	MI	RR	Age, Clinical CVD, Glucose, Race
	EPESE[14]	MI	OR	Age, Antihypertensive medication, BMI, Chest pain on exertion, Diabetes, Prevalent CHD at baseline, Smoking,
	FHS[11]	CVD	HR	Age, BMI, Diabetes mellitus, Smoking status, Total cholesterol
	FOS[42]	CVD	HR	Age, BMI, Cholesterol, HDL Cholesterol, HbA1c, Smoking, Diabetes
	PHS[32]	CHD	RR	Age, Alcohol intake, BMI, Current hypertension treatment, Diabetes, Exercise, Parental history if MI before 60y, Smoking status, Randomized aspirin treatment, Randomized beta-carotene treatment
	Other[44]	MI	HR	Age, Cholesterol, Left ventricular hypertrophy, PRA, Smoker, Urinary potassium, Urinary sodium

BMI: Body Mass Index; ES: effect size; HDL: High-Density Lipoprotein; HgbA1c: Glycosylated hemoglobin; PRA: Plasma Renin Activity.

[a]Abbreviation of study names: ARIC: Atherosclerosis Risk In Communities Study; CHS: Cardiovascular Health Study; EPESE: Epidemiologic Studies of the Elderly; FHS: Framingham Heart Study; FOS: Framingham Offspring Study; PHS: Physician's Health Study; WHI: Women's Health Initiative; WHS: Women's Health Study.

[b]Abbreviation of disease names: CHD: coronary heart disease; CVD: cardio vascular disease; MI: myocardial infarction.

[c]Abbreviation of ES types: HR: hazard ratio; OR: odds ratio; RR: relative risk.
