# Supplementary material for: Assessing Sex Differences in the Risk of Cardiovascular Disease and Mortality per Increment in Systolic Blood Pressure: A Systematic Review and Meta-Analysis of Follow-Up Studies in the United States
Source: PLoS One. 2017 Jan 25;12(1):e0170218. doi: 10.1371/journal.pone.0170218 (PMC5266379; doi:10.1371/journal.pone.0170218)
Supplement: S4 Table — (PDF) [file pone.0170218.s006.pdf]

S4 Table. Summary of Studies for CV Mortality.

	Study[d]	Disease[e]	ES type[f]	Adjustment Variables
Female				
	CHA[45]	CVD	HR	Age, BMI, BMI-square, Cholesterol, Cigarettes per day, ECG abnormality, Education, Race
	*CHA[46]	CVD	HR	Age, BMI, Cigarette smoking, Diabetes, Minor ECG abnormalities, Race, Serum cholesterol
	CHHS[13]	CHD	RR	Age, BMI, Diabetes status, Education year, Serum cholesterol, Smoking status
	EPESE[14]	CHD	OR	Age, Antihypertensive medication, BMI, Chest pain on exertion, Diabetes, Prevalent CHD at baseline, Smoking
	HERS[47]	CHD	HR	Age, Alcohol consumption, Baseline medications (statins, aspirin, angiotensin-converting enzyme inhibitors, alpha-blockers, beta-blockers, calcium channel blockers, diuretics, height, heart rate), Diabetes, Education year, Exercise, High-density-lipoprotein cholesterol level, Low-density lipoprotein cholesterol, Marital status, New York Heart Association level I-III heart failure class, Previous MI, Race, Serum triglycerides, Smoking, Serum creatinine, Treatment assignment, Waist-lip ratio
	RBS[48]	IHD	HR	Age, BMI, Cholesterol, Diabetes, Smoking
	WHI[43]	CVD	HR	Age, BMI, Diabetes mellitus, Current smoking, High cholesterol
Male				
	CHA[45]	CVD	HR	Age, BMI, BMI-square, Cholesterol, Cigarettes per day, ECG abnormality, Education, Race
	CHHS[13]	CHD	RR	Age, BMI, Diabetes status, Education year, Serum cholesterol, Smoking status
	ECHS[49]	IHD	OR	Age, Cholesterol, Cholesterol-square, Quetelet index, Quetelet index-square, Smoking current, Smoking past
	EPESE[14]	CHD	HR	Age, Antihypertensive medication, BMI, Chest pain on exertion, Diabetes, Prevalent CHD at baseline, Smoking,
	HAHS[50]	CVD	HR	Age, BMI, Cigarette smoking status, Hypertension in 1962/1966, Physical activity
	PGC[51]	CVD	HR	Age, BMI, BMI-square, Cigarettes per day, Plasma glucose, Serum cholesterol, Whitehall electrocardiogram ischemia
	PHS[52]	CVD	RR	Age, Alcohol intake, Aspirin use, BMI, DBP, Diabetes, Exercise, Multivitamin use, Tobacco use
	RBS[48]	IHD	HR	Age, BMI, Cholesterol, Diabetes, Smoking
	WCG[53]	CHD	HR	Age, Behavior type, Cholesterol, Cigarette smoking

BMI: Body Mass Index; DBP: Diastolic Blood Pressure; ECG: Electrocardiogram; ES: effect size; MI: myocardial infarction.

[d]Abbreviation of study names: CHA: Chicago Heart Association Detection Project in Industry; CHHS: Charleston Heart Study; ECHS: Evans County Heart Study; EPESE: Epidemiologic Studies of the Elderly; HAHS: Harvard Alumni Health Study; HERS: Heart and Estrogen/Progestin Replacement Study; PGC: Peoples Gas Company Study; PHS: Physician’s Health Study; RBS: Rancho Bernardo Study; WCG: Western collaborative group study; WHI: Women’s Health Initiative.

[e]Abbreviation of disease names: CHD: coronary heart disease; CVD: cardio vascular disease; IHD: ischemic heart disease.

[f]Abbreviation of ES types: HR: hazard ratio; OR: odds ratio; RR: relative risk.

[*]Study CHA[46] differs from CHA[45] in that it assesses a different age population.
