# Supplementary material for: Assessing Sex Differences in the Risk of Cardiovascular Disease and Mortality per Increment in Systolic Blood Pressure: A Systematic Review and Meta-Analysis of Follow-Up Studies in the United States
Source: PLoS One. 2017 Jan 25;12(1):e0170218. doi: 10.1371/journal.pone.0170218 (PMC5266379; doi:10.1371/journal.pone.0170218)
Supplement: S5 Table — (PDF) [file pone.0170218.s007.pdf]

S5 Table. Excluded publications due to overlapping studies.

No	Publication
1	PAN W-H, CEDRES LB, LIU K, DYER A, SCHOENBERGER JA, SHEKELLE RB, et al. Relationship of clinical diabetes and asymptomatic hyperglycemia to risk of coronary heart disease mortality in men and women. American journal of epidemiology. 1986;123(3):504-16.
2	Schoenbach VJ, Kaplan BH, Fredman L, Kleinbaum DG. Social ties and mortality in Evans County, Georgia. American journal of epidemiology. 1986;123(4):577-91.
3	Stamler J, Stamler R, Neaton JD. Blood pressure, systolic and diastolic, and cardiovascular risks: US population data. Archives of internal medicine. 1993;153(5):598-615.
4	Keil JE, Sutherland SE, Hames CG, Lackland DT, Gazes PC, Knapp RG, et al. Coronary disease mortality and risk factors in black and white men: results from the combined Charleston, SC, and Evans County, Georgia, heart studies. Archives of internal medicine. 1995;155(14):1521-7.
5	Klag MJ, Whelton PK, Randall BL, Neaton JD, Brancati FL, Ford CE, et al. Blood pressure and end-stage renal disease in men. New England Journal of Medicine. 1996;334(1):13-8.
6	Wilson PW, Hoeg JM, D'Agostino RB, Silbershatz H, Belanger AM, Poehlmann H, et al. Cumulative effects of high cholesterol levels, high blood pressure, and cigarette smoking on carotid stenosis. New England Journal of Medicine. 1997;337(8):516-22.
7	Franklin SS, Khan SA, Wong ND, Larson MG, Levy D. Is pulse pressure useful in predicting risk for coronary heart disease? The Framingham Heart Study. Circulation. 1999;100(4):354-60.
8	Sharrett AR, Sorlie P, Chambless LE, Folsom A, Hutchinson R, Heiss G, et al. Relative importance of various risk factors for asymptomatic carotid atherosclerosis versus coronary heart disease incidence: the Atherosclerosis Risk in Communities Study. American journal of epidemiology. 1999;149(9):843-52.
9	Kannel WB, Vasan RS, Levy D. Is the relation of systolic blood pressure to risk of cardiovascular disease continuous and graded, or are there critical values? Hypertension. 2003;42(4):453-6.
10	Yano Y, Stamler J, Garside DB, Daviglus ML, Franklin SS, Carnethon MR, et al. Isolated systolic hypertension in young and middle-aged adults and 31-year risk for cardiovascular mortality: the Chicago Heart Association Detection Project in Industry study. J Am Coll Cardiol. 2015;65(4):327-35.
